# Development of a Novel Double Antibody Sandwich Quantitative Enzyme-Linked Immunosorbent Assay for Detection of Porcine Epidemic Diarrhea Virus Antigen

**DOI:** 10.3389/fvets.2020.540248

**Published:** 2020-10-26

**Authors:** Baochao Fan, Jie Sun, Lin Zhu, Jinzhu Zhou, Yongxiang Zhao, Zhengyu Yu, Bing Sun, Rongli Guo, Kongwang He, Bin Li

**Affiliations:** ^1^Key Laboratory of Veterinary Biological Engineering and Technology Ministry of Agriculture, Institute of Veterinary Medicine, Jiangsu Academy of Agricultural Sciences, Nanjing, China; ^2^Jiangsu Key Laboratory for Food Quality and Safety-State Key Laboratory Cultivation Base of Ministry of Science and Technology, Nanjing, China; ^3^Jiangsu Co-infection Center for Prevention and Control of Important Animal Infectious Disease and Zoonoses, Yangzhou, China; ^4^Jiangsu Key Laboratory of Zoonoses, Yangzhou University, Yangzhou, China; ^5^School of Food and Biological Engineering, Jiangsu University, Zhenjiang, China

**Keywords:** PEDV, quantitative ELISA, antigen detection, intestinal and fecal samples, evaluation vaccine

## Abstract

Porcine epidemic diarrhea virus (PEDV) causes acute diarrhea and dehydration in sucking piglets with a high mortality rate. Here, we developed a double antibody sandwich quantitative enzyme-linked immunosorbent assay (DAS-qELISA) for detection of PEDV using a specific monoclonal antibody against PEDV N protein and anti-PEDV rabbit serum. Using DAS-qELISA, the detection limit of recombinant PEDV N protein and virus titer were approximately 1 μg/L and 10^2.0^ TCID_50_/ml, respectively. A total of 90 intestinal and 237 fecal samples were then screened for the presence of PEDV using DAS-qELISA and reverse transcriptase PCR (RT-PCR). DAS-qELISA had a high specificity of 98.1% and sensitivity of 93.5%. The accuracy rate between DAS-qELISA and RT-PCR was 95.7%. More importantly, the viral antigen concentrations remained unchanged before and after one inactivated vaccine preparation by using the DAS-qELISA. These results suggest DAS-qELISA could be used for antigen detection of inactivated vaccine samples and clinical samples. It is a novel method for diagnosing diseases and evaluation of the PEDV vaccine.

## Introduction

Porcine epidemic diarrhea virus (PEDV) is the causative agent of porcine epidemic diarrhea (PED), has caused huge economic losses to the swine industry all over the world (1, 2). Since December 2010, a large-scale outbreak of severe diarrhea has been re-emerged in swine farms of China, with 80–100% morbidity and 50–90% mortality in suckling piglets (3, 4). Increasing evidence suggests that this large-scale outbreak of diarrhea may be caused by highly virulent PEDV variants (5, 6). In May 2013, PED outbreaks suddenly emerged in the United States and spread rapidly throughout the country, as well as to Canada and Mexico. These outbreaks also caused high mortality rates in newborn piglets (7–9).

PEDV belongs to the *Alphacoronavirus* genus within the *Coronavirinae* subfamily of the *Coronaviridae* family (1, 10). PEDV, like other coronaviruses, contains a single-stranded, positive-sense RNA genome of about 28 kb. The virus produces a number of sub-genomic mRNAs, which encode for various non-structural and structural proteins within infected cells. The virus particles include spike (S) protein, envelope (E) protein, membrane (M) protein, and nucleocapsid (N) protein (11). The S protein is responsible for induction of neutralizing antibodies, specific receptor binding and cell membrane fusion (12). Moreover, according to the analysis of PEDV variability, the virus was found to be constantly mutating, and the amino acid mutations of PEDV pandemic strains were mainly located in the N-terminal domain of S1 (13).

PED cannot be accurately diagnosed based on clinical symptoms and histopathological lesions (14–17). Due to similarities between causative agents of diarrhea, differential diagnosis is necessary to identify PEDV in the laboratory (17, 18). The conventional detection method of PEDV is reverse transcription polymerase chain reaction (RT-PCR), and some RT-PCR alternatives have been developed, such as TaqMan-based real-time RT-PCR, reverse transcription loop-mediated isothermal amplification, and nanoparticle-assisted PCR assay (19–22). However, RT-PCR requires specialized laboratory equipment and experienced technicians. Moreover, optimization of the reaction system and conditions are laborious and complicated processes, which bear the risk of false-positive results due to laboratory contamination. An immunochromatographic assay was also developed to detect PEDV antigens. Unfortunately, this method has a low sensitivity, is not quantitative, and is unsuitable for high-throughput antigen detection (23). A new functionalized nanoparticle-based PCR method specific for PEDV was recently developed. However, as a new method, the reaction system and conditions are not yet optimized and could take time (24).

Enzyme-linked immunosorbent assay (ELISA) is a sensitive, specific, and convenient method for measuring macromolecular protein, bacteria and virus. The method uses stable reagents and inexpensive equipment, and the results are accurate and reproducible. In our study, we obtained monoclonal and polyclonal antibodies by immunizing mice and rabbits with purified recombinant N protein of PEDV variant strain AH2012/12 expressed in *Escherichia coli*. A double antibody sandwich quantitative ELISA (DAS-qELISA) was then established using a high-affinity monoclonal antibody (MAb) and horseradish peroxidase (HRP)-labeled rabbit polyclonal antibody as capture and detection antibodies, respectively. The assay demonstrated high sensitivity and specificity and could be used to detect PEDV antigens in diarrheal samples from piglets naturally infected with PEDV and inactivated vaccine samples.

## Materials and Methods

### Viruses, Cell Culture, and Preparation of PEDV N Protein

PEDV strain AH2012/12 (GenBank accession number: KU646831) was isolated and maintained in our laboratory, as previously described (25, 26). The Vero-81 (ATCC No. CCL-81) cell line was cultured in Dulbecco's Modified Eagle's Medium (DMEM; Thermo Fisher Scientific, MA, USA) supplemented with antibiotics (100 units/ml of penicillin, 100 μg /ml of streptomycin, and 0.25 μg /mL of amphotericin B; Thermo Fisher Scientific) and 10% heat-inactivated fetal bovine serum (FBS; Tianhang, China). Vero cells were maintained in DMEM containing 10 μg/ml trypsin and used to propagate PEDV. When obviously cytopathic effects were observed, the infected cell cultures were freeze-thawed, and cell debris was removed by centrifugation at 4,000 × *g* for 5 min at 4°C. The supernatant was collected and stored at −80°C until use. SP2/0 cells were obtained as described previously (27), and were maintained in RPMI 1,640 medium with 10% FBS.

Porcine transmissible gastroenteritis virus (TGEV), porcine rotavirus (RV), porcine reproductive and respiratory syndrome virus (PRRSV), classical swine fever virus (CSFV), porcine circovirus type 2 (PCV2), and porcine pseudorabies virus (PRV) were conserved in the laboratory and used to determine the specificity of DAS-qELISA.

The PEDV N protein was expressed and purified as described previously (28). Briefly, the N gene was amplified from PEDV strain AH2012/12 by RT-PCR with the following primers: forward: 5′-TTT*GGATCC*GCTTCTGTCAGTTTTCAGGATC-3′ (BamHI site underlined); reverse: 5′-CCG*CTCGAG*TTAATTTCCAGTATCGAAGATCTCG-3′ (XhoI site underlined). The amplicon was then digested with restriction enzymes BamHI and XhoI and ligated into the prokaryotic expression vector pET-28a, resulting in recombinant plasmid pET28a-N. *E. coli* BL21(DE3) cells carrying pET28a-N were grown in LB media containing kanamycin (25 mg/ml) at 37°C to A_600_ = 0.6 and then induced with 1 mM isopropyl-β-D-thiogalactopyranoside (IPTG) at 37°C for 5 h. The recombinant PEDV-N protein with 6 His-tag (rPEDV-N) was expressed as soluble protein after induction. And the protein was purified by Ni^2+^ affinity chromatography (Qiagen, Hilden, Germany) and verified by SDS-PAGE.

### Ethics Statement

All animal experiments were performed with the approval of the Jiangsu Academy of Agricultural Sciences Experimental Animal Ethics Committee (No. NKYVET 2015-0126). Efforts were made to minimize animal suffering and reduce the number of animals used.

### Recombinant N Protein Immunization Protocols of Animals

The immunization protocol followed conventional subcutaneous injection with slight modification (27). Briefly, 4 to 6-week-old female BALB/c mice were subcutaneously immunized with 0.1 mg of purified recombinant PEDV-N protein emulsified with complete Freund's adjuvant (Sigma-Aldrich, St. Louis, MO, USA), followed by two subcutaneous immunizations with 0.1 mg of rPEDV-N in incomplete Freund's adjuvant every 4 weeks.

Four female adult (12-week-old) rabbits were also immunized according to the mice immunization protocol. However, the dose of immunization was ten-fold higher than that used in mice. Blood samples were collected from tail vein of mice or ear vein of rabbits on the 7th day after each booster immunization. The antibody titers were detected by indirect ELISA.

### Preparation and Identification of Monoclonal Antibodies (MAbs) Against PEDV N

Three days prior to cell fusion, mice were boosted with 0.1 ml rPEDV-N solution (0.5 mg/ml). Mice were then bled and serum samples were collected and the antibody titers against rPEDV-N were tested by ELISA with rPEDV-N as the coating antigen. The mouse with the highest antibody titer was euthanized and its spleen was collected. The fusion of B lymphocytes with mouse myeloma cells was carried out as described previously (29). The resulting hybridoma cells were plated in 96-well plates and cultured in HAT selection medium (DMEM containing 20% FBS, 100 mg/ml streptomycin, 100 IU/ml penicillin, 100 mM hypoxanthine, 16 mM thymidine, and 400 mM aminopterin). The antibody titers of hybridoma supernatants against N protein were screened by indirect ELISA. Positive clones were subcloned and rescreened. MAbs with high antibody titers were then purified from ascites using the octanoic acid/saturated ammonium sulfate precipitation method and subsequently purified by protein G-sepharose columns. Isotypes of obtained MAbs were determined by using a commercially mouse MAb isotyping kit (Zymed Laboratories, Carlsbad, CA, USA).

### Rabbit Polyclonal Antibody Preparation

Seven days after the final injection, the rabbit with the highest antibody titer was anesthetized by intraperitoneal injection of 10% chloral hydrate to collect whole blood and obtain the serum. The rabbit serum antibody titer reached 1:243,000. The antibody was purified from the serum by using octanoic acid-saturated ammonium sulfate precipitation and protein A-sepharose columns, and was desalinated over a Sephadex G-25 column. The purified polyclonal antibody was stored at −70°C. The antibody titers were assayed by indirect ELISA.

### Selection of Antibody Pairs

The purified rabbit polyclonal antibody was labeled with HRP (Sigma-Aldrich). Each prepared MAb against PEDV N protein was coated onto wells of a 96-well microtiter plate (Costar, Corning, NY, USA). rPEDV-N (1μg/ml) or positive sample (PEDV culture supernatant) was used as the sandwich antigen, and HRP-labeled rabbit polyclonal antibody was used as the detection antibody to perform DAS-ELISA for antibody pairing. As a negative control, the sandwich antigen was replaced with phosphate-buffered saline (PBS). The test results are expressed as OD_450_ values. The best antibody pairs were obtained according to the recorded result.

### Establishment and Optimization of DAS-qELISA

We next selected the best combination of capture mouse MAbs and detection polyclonal antibody for PEDV antigen-capture ELISA. Briefly, microplates were coated with 100 μl/well of each capture MAb at a concentration of 2 μg/ml at 4°C overnight. After blocking, serially diluted PEDV-infected culture supernatants or uninfected controls were added into the wells in duplicate, and the plates were placed at 25°C in a dark environment for 45 min. After washing, HRP-labeled polyclonal antibodies were added at a working concentration, and plates were incubated at 25°C for 45 min. Then, the wells were washed with PBS with 0.5% Tween 20, and added 3,3′,5,5′-Tetramethylbenzidine (TMB) solution. Fifteen minutes later at 25°C, sulfuric acid (2 M, 100 μl/well) was added to stop the reaction, and the absorbance at OD_450_ was measured.

### DAS-qELISA Positive and Negative Cut-Off Values

A total of 40 PEDV negative fecal and intestinal samples were obtained from healthy piglets. These samples were diluted with PBS (0.01 M, pH 7.2) to obtain a 10% suspension (v/v), clarified by centrifugation at 2,000 g for 10 min, the supernatant was treated with 1% Triton X-100 and 0.3% tri-n-butyl phosphate (TNBP) for 2 h at room temperature (RT) to inactivate the virus (30), and then was detected by the established DAS-qELISA with the determined optimal conditions. The cut-off value at OD_450_ to identify a PEDV-positive sample was calculated based on the following formula: positive and negative cut-off value = negative sample mean + 2 standard deviations (mean + 2SD).

### Detection Limit and Cross-Reaction of DAS-qELISA

To evaluate the sensitivity of DAS-qELISA, plates were coated with mouse MAbs and incubated overnight at 4°C. Then, the plates were blocked with PBS containing 2% bovine serum albumin and 100 μl of 0, 1.0, 2.0, 4.0, 8.0, 16.0, and 32.0, μg/L diluted rPEDV-N protein standard with PBS was added, then the plates were incubated for 45 min at 25°C. After washing, 100 μl diluted rabbit HRP-labeled anti-PEDV-N polyclonal antibody was then added, and plates were incubated at 25°C for 45 min. Plates were then washed, and 100 μl of TMB solution was added. After incubating at 25°C in the dark for 15 min, sulfuric acid (2 M, 100 μl/well) was added, and the absorbance was measured at 450 nm to determine the standard curve. In order to determine the detection limit of virus titer. The PEDV-infected culture supernatant was centrifuged by using one ultrafiltration membrane reactor with molecular weight cutoff 100 kDa (Merck KGaA, Darmstadt, Germany) to remove free N protein. The purified virus was diluted with the same volume of medium and titrated by TCID_50_. Acquired10^5.74^TCID_50_/ml purified PEDV solution was inactivated by treatment with 1% Triton X-100 and 0.3% TNBP for 2 h at RT, and was serially diluted 2- or 10-fold, assayed by DAS-qELISA, and absorbance measured by the ELISA plate reader.

To evaluate the specificity of DAS-qELISA, suspensions of TGEV, RV, PRRSV, CSFV, PCV2, and PRV were selected for testing. PEDV-positive viral suspensions and PEDV-negative samples from non-infected (mock) cell debris were also evaluated by DAS-qELISA.

### Duplicability Test

The duplicability test was carried out as described previously (31). Briefly, the intra-batch assay was determined by detecting each sample in microplates coated with capture MAb by DAS-qELISA in three parallel wells. These samples were also detected by DAS-qELISA in microplates coated with different batches of capture MAbs for inter-batch assay. All tests were repeated three times. Intra- and inter-assay coefficients of variation (%CV) were calculated by the following formula: %CV = (standard deviations (SD)/mean OD_450_ of eight samples) ×100%.

### Comparison of DAS-qELISA and RT-PCR

A total of 90 intestinal and 237 fecal samples obtained from different pig farms were processed as described above, and were screened for the presence of PEDV using DAS-qELISA and RT-PCR. The sensitivity, specificity, and accuracy were calculated by the following formulas: sensitivity = true positive/(true positive + false negative) ×100%; specificity = true negative/(true negative + false positive) ×100%; accuracy = (true positive + true negative)/(true positive + false positive + true negative + false negative) ×100%. The agreement between RT-PCR and DAS-qELISA techniques was measured with the kappa statistic value (32).

### Preparation of Vaccine Antigens and Formulation of Inactivated PEDV Vaccine

The supernatant of Vero cells containing PEDV strain AH2012/12 was obtained as described above. And the virus titer was tested and adjusted to 10^6.0^ TCID_50_/ml. Then it was inactivated with 0.1% formaldehyde at 37°C for 48 h. A vaccine was prepared by mixing the inactivated PEDV vaccine antigen in an equal volume of Montanide ISA 201 adjuvant (Seppic, Paris, France).

## Results

### PEDV N Protein Expression in *E. coli*

**Figure 1:**
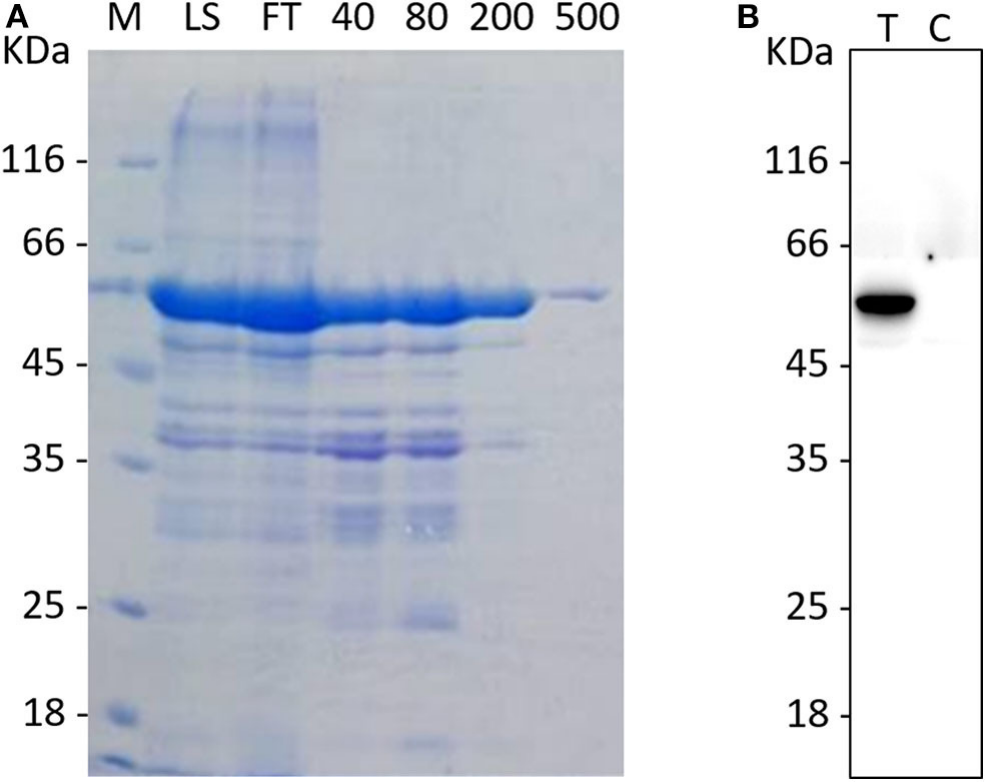
The purification and identification of the recombinant N protein. **(A)** The purification of the rPEDV-N protein. The MK lane represents a marker, the LS lane represents injection sample, and the FT lane represents the outflow liquid with not binding. The 40, 80, 200, and 500 lanes represent the eluents with imidazole concentration of 40, 80, 200, and 500, respectively. **(B)**The western blot identification of rPEDV-N protein. The T lane represents purified rPEDV-N protein, and the C lane represents the lysates of *E. coli* BL21 cells.

Recombinant vectors were transformed into competent *E. coli* BL21 cells and induced with 1 mM IPTG at 37°C for 5 h to express the recombinant protein. The purified protein is shown in Figure 1A; the molecular weight of the recombinant protein was about 50 kDa, which corresponded with the molecular weight of fusion protein His-N. The correct corresponding protein band was confirmed by western blot using anti-N monoclonal antibody (Figure 1B).

### Preparation and Characterization of MAbs

Antibody-positive subclones were obtained by the limited dilution method. Ten days after the test, positive clones were continuously isolated and selected by the limited dilution method for subcloning. A total of 37 hybridoma cell lines capable of secreting MAbs against PEDV N protein were obtained and numbered #1–37. All hybridomas producing antibodies were detected by indirect ELISA with 0.5 as the cut-off value. A 96-well ELISA plate was coated with purified rPEDV-N at 1 μg/ml. Among the 37 MAbs, 23 had antibody titers >1:1,024,000, 5 had titers ranging from 1:512,000 to 1:1,024,000, 4 has titers between 1:64,000 and 1:128,000, and 5 had titers <1:64,000. Antibody subtypes were also determined using the isotype detection kit. Among the 37 MAbs, 30 were IgG_1a_, 3 were IgG_2a_, 3 were IgG_2b_, and 1 was IgM.

### Paired Antibody Selection

We selected 16 MAbs that had the highest antibody titers for antibody pairing. The data are shown in Table 1. Seven MAbs had positive sample OD_450_ values of more than 2.1 times negative sample OD_450_ values, which were judged as positive. MAb #16 had the highest OD_450_ value for positive samples, rPEDV-N and virus supernatant, and the lowest OD_450_ value for negative samples. Therefore, MAb #16 was selected for development of DAS-qELISA. MAb #16 was prepared from hybridoma cell line #16, which was subtype IgG_1a_ and had a titer of >1:1,024,000.

**Table 1:** The selection of pairing antibodies.

**mAbs No.(#)**	**Mean OD_**450**_**
	**rPEDV-N protein (1 μg)**	**Viral supernatant**	**Negative**
2	3.877	0.745	0.445
3	3.766	0.553	0.411
5	3.829	1.5	0.374
7	3.822	0.562	0.477
9	3.884	1.45	0.413
13	3.818	1.249	0.446
14	3.839	0.724	0.432
16	3.894	3.69	0.223
19	3.904	0.877	0.252
20	3.348	0.576	0.317
21	3.838	0.559	0.419
22	3.343	0.625	0.358
23	3.89	0.51	0.334
25	3.359	0.824	0.251
26	3.868	1.263	0.228
27	3.369	0.463	0.305

### Development of DAS-qELISA

MAb #16 and rabbit HRP-labeled polyclonal antibody were determined as the optimal capture and detector antibodies by indirect ELISA, respectively. Optimum reaction conditions of the antigen-capture assay was conducted by a checkerboard analysis of serial dilutions of capture and detection antibodies. And the results showed that the optimal concentrations for capture by MAb #16 and for detection by HRP-labeled polyclonal antibody were both 2 μg/mL. Forty PEDV-negative fecal samples were used to determine the cut-off value. The mean (X) was 0.168 and the standard deviation (SD) was 0.017, thus the critical value (X + 2SD) was 0.201, which was used to define negativity and positivity.

### Specificity of DAS-qELISA

The specificity of DAS-qELISA was assessed by testing culture supernatants of six other viruses: TGEV, RV, PRRSV, CSFV, PCV2, and PRV. As shown in Table 2, only PEDV-infected cell culture supernatants presented a positive signal, and no positive results or cross-reactivity was observed for the other viruses. These results indicated that the DAS-qELISA method was specific for PEDV detection.

**Table 2:** The results of specificity assay.

**Virus**	**PEDV**	**TGEV**	**RV**	**PRRSV**	**CSFV**	**PCV2**	**PRV**
Mean OD_450_ values	1.086	0.113	0.111	0.112	0.1	0.101	0.11
PEDV-N concentration(μg/L)	26.45	−0.53	−0.64	−0.59	−1.1	−0.94	−0.65

*The different pathogens were inactivated by treatment with 1% Triton X-100 and 0.3% TNBP for 2 h at RT, and then detected by the DAS-qELISA at optimum conditions*.

### Detection Limit of PEDV DAS-qELISA

**Figure 2:**
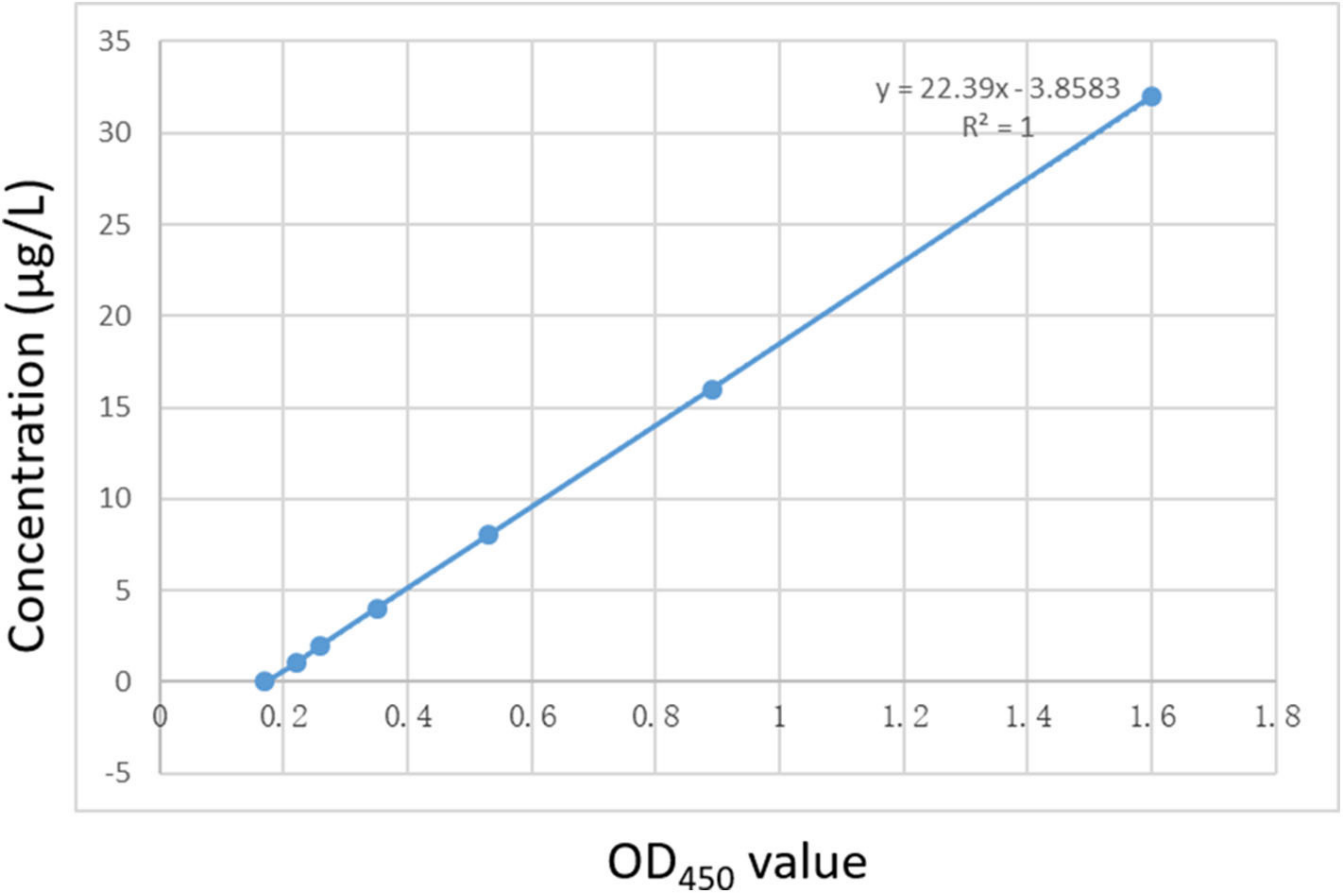
The standard curve of the DAS-qELISA. Two-fold serially diluted standard rPEDV-N protein with concentrations of 32, 16, 8, 4, 2, 1, and 0 μg/L was assayed using DAS-qELISA with two replicates per concentration. The standard curve was calculated using a linear relationship between the OD_450_ values and concentrations.

PEDV DAS-qELISA was assayed by using 2-fold serially diluted standard rPEDV-N protein with concentrations of 32, 16, 8, 4, 2, 1, and 0 μg/L. The standard curve was obtained as follows: Y = 22.39X−3.8,583, *R*^2^ = 1.0. The detection limit of rPEDV-N protein was approximately 1 μg/L (Table 3). The standard curve is shown in Figure 2.

**Table 3:** The assay of standard rPEDV-N protein by using DAS-qELISA kit.

**rPEDV-N concentration(μg/L)**	**Mean OD_**450**_ values**
32	1.60
16	0.89
8	0.53
4	0.35
2	0.26
1	0.22
0	0.17

*two-fold serially diluted standard rPEDV-N protein with concentrations of 32, 16, 8, 4, 2, 1, and 0 μg/kgL was assayed by using DAS-qELISA*.

The detection limit of virus titer was also assayed by using PEDV DAS-qELISA. A 10^5.74^ TCID_50_/ml purified PEDV virus was serially diluted 10-fold (Table 4) and 2-fold (Table 5), respectively. The value of 10^2.03^ TCID_50_/ml of virus supernatant was above the critical value of 0.229, indicating that the detection limit of DAS-qELISA was about 10^2.0^ TCID_50_/ml.

**Table 4:** The results of DAS-qELISA of 10-fold dilution of purified PEDV virus.

**Dilution fold of virus**	**DAS-qELISA**		
	**TCID_**50**_/ml**	**Mean OD_**450**_ values**	**Concentration (μg/L)**
1:10	10^4.74^	3.065	127.25
1:100	10^3.74^	3.091	128.39
1:1,000	10^2.74^	1.557	61.18
1:10,000	10^1.74^	0.207	2.03
1:100,000	10^0.74^	0.176	0.67
CON	0	0.165	0.19

*The 10^5.74^TCID_50_/ml purified virus was presently inactivated and serially diluted 10-fold, then was assayed by DAS-qELISA*.

**Table 5:** The results of DAS-qELISA of 2-fold dilution of purified PEDV virus.

**Dilution fold of virus**	**DAS-qELISA**		
	**TCID_**50**_/ml**	**Mean OD_**450**_ values**	**Concentration (μg/L)**
1:10	10^4.74^	3.065	127.25
1:20	10^4.44^	3.303	137.68
1:40	10^4.14^	3.321	138.47
1:80	10^3.84^	3.189	132.69
1:160	10^3.54^	2.912	120.55
1:320	10^3.23^	2.552	104.78
1:640	10^2.93^	1.866	74.72
1:1,280	10^2.63^	0.901	32.44
1:2,560	10^2.33^	0.296	5.93
1:5,120	10^2.03^	0.229	2.99
1:10,240	10^1.73^	0.193	1.43
CON	0	0.165	0.19

*The 10^5.74^TCID_50_/ml purified virus was presently inactivated and serially diluted 2-fold, then was assayed by DAS-qELISA*.

### Reproducibility of DAS-qELISA

The results of duplicability testing are shown in Tables 6, 7. The %CV of intra- and inter-batch duplicability tests was between 2.4 and 7.8% and between 3.4 and 8.4%, respectively. These results indicated that the developed DAS-qELISA was adequate for PEDV detection.

**Table 6:** The results of intra-batch duplicability test.The results of intra-batch duplicability test.

**Assay time**	**No. of PEDV positive fecal samples**							
	**1**	**2**	**3**	**4**	**5**	**6**	**7**	**8**
First	17.4	154.2	55.0	128.0	35.7	70.6	35.4	75.4
Second	16.2	144.6	50.1	124.8	32.1	75.4	36.8	75.1
Third	18.7	174.2	50.7	139.7	30.7	67.6	32.5	79.1
X	17.4	157.7	51.9	130.8	32.8	71.2	34.9	76.5
SD	1.0	12.3	2.2	6.4	2.1	3.2	1.8	1.8
CV	5.9%	7.8%	4.2%	4.9%	6.4%	4.5%	5.1%	2.4%

*Eight pre-treated fecal samples were assayed by DAS-qELISA. X, mean N protein content of each sample; SD, standard deviation; CV, coefficients of variation*.

**Table 7:** The results of inter-batch duplicability test.

**Batch number**	**No. of PEDV positive fecal samples**							
	**1**	**2**	**3**	**4**	**5**	**6**	**7**	**8**
First	17.5	176.0	56.3	124.3	28.6	84.7	36.4	75.9
Second	15.4	162.9	55.3	123.9	24.1	74.2	36.1	71.0
Third	15.7	143.0	47.1	136.4	26.7	86.9	39.7	70.3
X	16.2	160.6	52.9	128.2	26.5	81.9	37.4	72.4
SD	0.9	13.6	4.1	5.8	1.8	5.5	1.6	2.4
CV	5.7%	8.4%	7.8%	4.5%	7.0%	6.8%	4.4%	3.4%

*Eight pre-treated fecal samples were assayed by DAS-qELISA. X, mean N protein content of each sample; SD, standard deviation; CV, coefficients of variation*.

### Field Sample Detection

A total of 90 intestinal and 237 fecal samples were screened for the presence of PEDV using DAS-qELISA and RT-PCR (Table 8). In intestinal samples, 68 of the 90 field samples were determined to be positive by using DAS-qELISA, whereas 65 samples were positive by RT-PCR. Three samples gave discordant results, which were PEDV-positive by DAS-qELISA but PEDV-negative by RT-PCR. In fecal samples, a total of 93 samples were positive in both DAS-qELISA and RT-PCR and 133 were negative in both tests. Eleven samples gave discordant results, which were PEDV-negative by DAS-qELISA but PEDV-positive by RT-PCR. Overall, DAS-qELISA was found to have 98.1% specificity (155/158) and 93.5% sensitivity (158/169) relative to RT-PCR. And the accuracy of these two detection methods was [(87 + 226)/327] = 95.7%. In addition, the kappa values were 0.914 and 0.905 by examining fecal and intestinal samples respectively, suggesting an almost perfect agreement between the DAS-qELISA and RT-PCR methods.

**Table 8:** Comparison of RT-PCR and DAS-qELISA for the detection of PEDV in **(A)** intestinal and **(B)** fecal samples.

**RT-PCR**	**DAS-qELISA**		
	**Positive**	**Negative**	**Total**
A	*K* = 0.914		
Positive	65	0	65
Negative	3	22	25
Total	68	22	90
B	*K* = 0.905		
Positive	93	11	104
Negative	0	133	133
Total	93	144	237

### Vaccine Sample Detection

Viral antigen content changes before and after vaccine preparation were also tested by using DAS-qELISA. As shown in Table 9, the samples of 10, 50, and 100-fold dilution multiples exceeded the test limit of DAS-qELISA, and the result was not credible. Thus, the concentration of the virus stock solution was about 14,000 μg/L. After inactivation and preparation into a vaccine, the concentration of the vaccine was about 7,000 μg/L. Considering the vaccine was prepared by mixing an equal volume of killed antigen and adjuvant, viral antigen concentrations likely remained unchanged before and after vaccine preparation.

**Table 9:** The detection of antigen concentration of PEDV viral supernatant and vaccine by using DAS-qELISA.

**No**.	**Viral supernatant**			**Vaccine**		
**Dilution**	**Mean OD_**450**_**	**Concentration**	**Final**	**Mean OD_**450**_**	**Concentration**	**Final**
**factor**	**values**	**(μg/L)**	**concentration**	**values**	**(μg/L)**	**concentration**
			**(μg/L)**			**(μg/L)**
10	3.23	42.81	428	2.91	38.51	385
50	3.20	42.41	2,120	2.63	34.74	1,737
100	3.26	43.22	4,321	2.21	29.09	2,909
1,000	**1.08**	**13.89**	**13,892**	**0.70**	**8.78**	**8,781**
2,000	**0.56**	**6.90**	**13,796**	**0.32**	**3.67**	**7,340**
4,000	**0.32**	**3.67**	**14,679**	0.12	0.98	3,919

*The formula of standard sample was Y = 13.451x−0.6,344, and R^2^ = 0.9,994. The data marked in bold represents the results in the confidence interval*.

## Discussion

As a re-emerged disease, PEDV has caused huge economic losses to the swine industry all over the world. Early and rapid diagnosis of PEDV is very important to prevent and control the spread of this disease. At present, RT-PCR is the main technology for the diagnosis of microbial infections. However, this method has some shortcomings, such as the need for expensive specialized equipment, the instability of RNA samples, and the possible contamination. Other rapid tests are therefore needed to facilitate diagnosis. In this study, we developed a DAS-qELISA, which could be used for quantitative detection of viral antigens, by using one mouse MAb and rabbit polyclonal antibody as capture and detection antibodies, respectively. The described assay could detect up to 1 μg/L of PEDV N protein and 10^2.0^ TCID_50_/ml virus stock. No cross-reactivity with other similar causative agents of diarrhea and important pig pathogens, such as TGEV, RV, PRV, PRRSV, CSFV, PCV2 and PRV, was observed. Furthermore, the results of field sample detection reveal a positive coincidence between DAS-qELISA and RT-PCR. This newly developed DAS-qELISA with high sensitivity and specificity could be used as an effective method for the diagnosis of PEDV infection in pigs.

In a previous study, a DAS-ELISA to detect PEDV antigen was developed by using MAbs to M protein of PEDV classical strain CV777 (33). However, in recent years, highly virulent PEDV variants have become the main pathogens of porcine diarrheal diseases, which show large genetic differences compared with classical strains, such as CV777 and DR13 (3, 5). Recently, two studies reported similar ELISA methods for detecting pathogens using antibodies to S protein as capture antibodies (34, 35). Although the use of antibody against S could improve the specificity of the detection method, S protein, as one of the viral proteins with the highest mutation rate, might reduce the detection efficiency of the mutant virus. In this study, because of the high homology of N protein amino acid sequences from different PEDV strains, and the high immunogenicity of this protein, N protein seems to be a suitable antigen marker for the diagnosis of PEDV infection. Therefore, in this study, we used the N protein of the PEDV variant strain AH2012/12 as an immunogen to obtain MAbs and polyclonal antibodies. In addition, although the N protein is located inside the virus particles, we used reagents to inactivate the virus, which may enable the antibody to pass through the envelope and react with the internal N protein.

A total of 37 MAbs against PEDV-N were produced by immunizing mice with rPEDV-N. The reaction profile of each antibody was characterized by indirect ELISA and IFA. All 37 MAbs were found to be specific to PEDV-N (data not shown). These MAbs allowed for the selection of an optimal MAb for constructing a DAS-qELISA with HRP-labeled rabbit polyclonal antibody. We found that the matched pair of MAb #16 as the capture antibody exhibited the highest sensitivity (binding) to PEDV-infected culture supernatant. Moreover, we generated a polyclonal antibody and used it as the detection antibody. Based on the ability of polyclonal antibody to bind to multiple antigenic epitopes, this method can reduce the missed detection rate and increase the sensitivity of the assay. By using MAb #16 and rabbit HRP-labeled polyclonal antibody, the detection limit of DAS-qELISA was as low as 1 μg/L with rPEDV-N protein and 10^2.0^ TCID_50_/ml with PEDV culture supernatants. Moreover, to determine specificity of this DAS-qELISA detection method, other porcine gastroenteric diseases (i.e., TGEV, RV, and PRV) and several important porcine pathogens (i.e., PRRSV, CSFV, and PCV2), which are likely to cause co-infection in pigs, were examined. No cross-reaction was observed with these viruses, suggesting that this method has a high level of specificity for PEDV.

We next tested fecal and intestinal samples by using DAS-qELISA and RT-PCR. When examining intestinal samples, only three samples gave discordant results, which were PEDV-positive by DAS-qELISA but PEDV-negative by RT-PCR. This disagreement might be due to the presence of PCR inhibitors and nucleic acid-degrading substances in intestinal samples, and they were retained in extracted nucleic acids, thus affecting the accuracy of PCR. It may also be due to poor quality of the collected samples, such as autolysis of tissues stored at room temperature for a long time. In the 237 fecal samples, 11 samples gave discordant results, which were PEDV-negative by DAS-qELISA but PEDV-positive by RT-PCR. The DAS-qELISA test may fail to detect antigens with very low viral titers in fecal samples. In this study, some PEDV-positive intestinal samples could not be detected by using RT-PCR, and this result was also observed by Sozzi et al. (36). Overall, the kappa value of these two different methods were both >0.90, suggesting a very high consistency between the two methods.

We also tested viral antigen contents before and after vaccine preparation by using DAS-qELISA. After inactivation and emulsification with the adjuvant, the antigen of the prepared PEDV vaccine could also be tested. Viral antigen concentrations were not changed before and after vaccine preparation. This result revealed that our DAS-qELISA method could be used for the detection of vaccine samples.

In summary, we developed a DAS-qELISA method for detection of PEDV antigen by using a specific MAb against PEDV N protein and anti-PEDV-N rabbit serum. This method has a high specificity and sensitivity, and the accuracy rate between DAS-qELISA and RT-PCR was 95.7%. These results indicate that the DAS-qELISA method could be used for diagnosing diseases caused by PEDV.

## Data Availability Statement

The original contributions presented in the study are included in the article/supplementary material, further inquiries can be directed to the corresponding author/s.

## Ethics Statement

The animal study was reviewed and approved by Jiangsu Academy of Agricultural Sciences Experimental Animal Ethics Committee.

## Author Contributions

BL and KH designed the experiment. BF and BL wrote the manuscript. Clinical samples were collected by BF, ZY, and LZ. JZ, YZ, JS, BS, and RG detected the samples by ELISA. BF, BL, and RG analyzed the data. All authors took part in discussion and interpretation of results. All authors read, advised, and approved the final manuscript.

## Conflict of Interest

The authors declare that the research was conducted in the absence of any commercial or financial relationships that could be construed as a potential conflict of interest.
